# Comparison of the Acute Effects of Different Pacing Sites on Cardiac Synchrony and Contraction Using Speckle-Tracking Echocardiography

**DOI:** 10.3389/fcvm.2021.758500

**Published:** 2021-11-11

**Authors:** Huilin Xie, Xueying Chen, Yanan Wang, Yufei Cheng, Yingjie Zhao, Yang Liu, Yu Liu, Zhenyi Ge, Haiyan Chen, Xianhong Shu

**Affiliations:** ^1^Department of Echocardiography, Zhongshan Hospital, Fudan University, Shanghai, China; ^2^Department of Cardiology, Zhongshan Hospital, Fudan University, Shanghai, China; ^3^Shanghai Institute of Cardiovascular Diseases, Fudan University, Shanghai, China; ^4^Shanghai Institute of Medical Imaging, Fudan University, Shanghai, China

**Keywords:** cardiac synchrony, physiological pacing, echocardiography, His bundle pacing, left bundle branch pacing

## Abstract

**Background:** Cardiac pacing in patients with bradyarrhythmia may employ variable pacing sites, which may have different effects on cardiac function. Left bundle branch pacing (LBBP) is a new physiological pacing modality, and the acute outcomes on cardiac mechanical synchrony during LBBP remain uncertain. We evaluated the acute effects of four pacing sites on cardiac synchrony and contraction using speckle-tracking echocardiography, and comparisons among four different pacing sites were rare.

**Methods:** We enrolled 21 patients with atrioventricular block or sick sinus syndrome who each sequentially underwent acute pacing protocols, including right ventricular apical pacing (RVAP), right ventricular outflow tract pacing (RVOP), His bundle pacing (HBP), and left bundle branch pacing (LBBP). Electrocardiograms and echocardiograms were recorded at baseline and during pacing. The interventricular mechanical delay (IVMD), the standard deviation of the times to longitudinal peak strain during 17 segments (PSD), and the Yu index were used to evaluate ventricular mechanical synchrony. Layer-specific strain was computed using two-dimensional speckle tracking technique to provide in-depth details about ventricular synchrony and function.

**Results:** Left ventricular ejection fraction (LVEF) and tricuspid annulus plane systolic excursion (TAPSE) were significantly decreased during RVAP and RVOP but were not significantly different during HBP and LBBP compared with baseline. RVAP and RVOP significantly prolonged QRS duration, whereas HBP and LBBP showed non-significant effects. IVMD and PSD were significantly increased during RVAP but were not significantly different during RVOP, HBP, or LBBP. LBBP resulted in a significant improvement in the IVMD and Yu index compared with RVAP. No significant differences in mechanical synchrony were found between HBP and LBBP.

**Conclusion:** Among these pacing modalities, RVAP has a negative acute impact on cardiac synchrony and contraction. HBP and LBBP best preserve physiological cardiac synchrony and function.

## Introduction

Cardiac pacing, an effective therapy for patients with bradyarrhythmia, has multiple modalities, including right ventricular apical pacing (RVAP) (1), right ventricular outflow tract pacing (RVOP) (2), His bundle pacing (HBP) (3), and left bundle branch pacing (LBBP) (4). RVAP is the traditional mode and has the advantage of long-term lead stability and ease of access, but it impairs left ventricular (LV) function due to asynchronous electrical activation (5). As an alternative, RVOP allows more physiological stimulation; however, a previous study indicated that the long-term clinical outcomes of RVOP were not superior to those of RVAP (6). HBP activates the intrinsic His-Purkinje conducting system, thus preserving synchronized ventricular contraction (7); it is limited by high and unstable pacing thresholds, long implantation times, and high dislodgement rates (8). LBBP, a recent form of His-Purkinje system pacing introduced by Huang et al. in 2017 (4), is considered to provide physiological activation. In this modality, the block position is circumvented and the left bundle branch (LBB) area is directly activated to synchronize LV contraction with a low and stable threshold. However, the right bundle branch is ignored and right bundle branch block (RBBB) has occurred; whether LBBP contributes to ventricular mechanical dyssynchrony remains uncertain. Long-term cardiac systolic asynchrony leads to remodeling of the cardiac contraction and electrophysiological characteristics and further aggravates the electrical and mechanical dyssynchrony, increasing the risk of atrial fibrillation and heart failure (9).

This study evaluated the acute effects of different pacing sites on cardiac synchrony and contraction in patients with atrioventricular block (AVB) or sick sinus syndrome (SSS) using echocardiography.

## Methods

### Study Population

Between March and June 2018, we prospectively enrolled consecutive patients with AVB or SSS who were scheduled for pacemaker implantation. The inclusion criteria were: (1) no history of pacemaker implantation, (2) no pregnancy, (3) at least 18 years of age, (4) New York Heart Association (NYHA) classification I or II. Patients were excluded for the following conditions: (1) severe valvular regurgitation, (2) recent acute myocardial infarction, (3) a history of cardiac surgery or atrioventricular node ablation, (4) poor acoustic window condition, (5) confirmed infra-His bundle block, or (6) the presence of severe chronic diseases. The study conformed with the 1975 Declaration of Helsinki, and the protocol was approved by the Zhongshan Hospital Ethics Committee. All patients provided their written informed consent to participate in the study.

### Pacing Procedure

**Figure 1:**
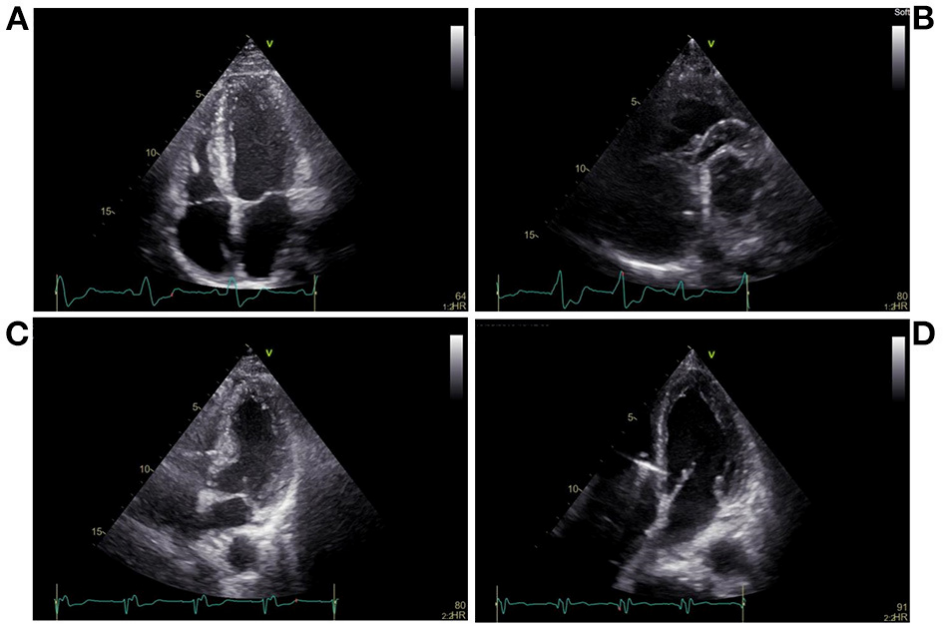
Echocardiography during pacing at different sites. **(A)** Right ventricular apical pacing, **(B)** Right ventricular outflow tract pacing, **(C)** His bundle pacing, **(D)** Left bundle branch pacing.

The pacing procedures were performed in a cardiac catheterization laboratory. Twelve-lead electrocardiogram (ECG) and intracardiac electrograms were simultaneously displayed and continuously recorded during all pacing interventions on a multichannel Bard Electrophysiology Lab System recorder (Bard, Haverhill, MA, USA). A catheter with a 6-Fr quadripolar electrode was inserted *via* the right external jugular vein; the electrodes were positioned within the right ventricular (RV) apex (RVA) and RV outflow tract (RVOT) (Figures 1A,B). For HBP, a preformed sheath (C315 HIS, Medtronic, Minneapolis, MN USA) was inserted *via* the right external jugular vein and placed in the region near the tricuspid valve septal leaflet. A Select Secure pacing lead (Model 3830, 69 cm, Medtronic) was delivered along the sheath with its distal part beyond the tip of the sheath for HBP recording (Figure 1C). For LBBP, the lead was twisted deeply through the ventricular septum from the RV septum to the endocardium of the LV septum to activate the LBB region (Figure 1D). According to the intracardiac electrograms, the LBB potential gradually appeared and increased as the electrode was screwed in, and the QRS morphology was gradually transformed from LBBB to RBBB. The interval between the LBB potential and ventricular activation was shorter than between the His bundle potential and ventricular activation. The imaging characteristics of LBBP showed that the pacing site was in the ventricular septum. The left or right anterior oblique projection was used to assist in identifying catheter positions; endocardial ECG was utilized to confirm these positions.

Each pacing mode was separated by a 10-min washing-out interval. In all patients, the pacing sequence ended with LBBP, and the lead was left in place after LBBP. During each procedure, the atrial lead was implanted in the right atrium appendage. Both the atrial and ventricular leads at the four pacing sites were connected to the programmer (Medtronic 2290) in DDD mode, with an AV delay of 150 ms and a pacing output of 3.5 V/0.5 ms during unipolar configuration.

### ECG and Echocardiography

ECGs and echocardiography were performed at baseline and during each pacing modality. During each session, patients were kept in the left lateral decubitus position with the ECG connected. Two-dimensional echocardiography was performed, according to current guidelines, using a Vivid E95 scanner (GE Vingmed Ultrasound, Horten, Norway) equipped with an M5S probe (4.0-MHz transducer) having frame rates higher than 40 fps (10). LV end-diastolic volume (LVEDV), LV end-systolic volume (LVESV), and tricuspid annulus plane systolic excursion (TAPSE) were derived from M-mode images. The LV ejection fraction (LVEF) was measured using the biplane Simpson's method, per guideline recommendations (11). To evaluate interventricular dyssynchrony, we measured the interventricular mechanical delay (IVMD) as the time interval between the beginning of QRS and the beginning of the systolic waves of aortic and pulmonary ejections, using conventional Doppler (12). Intraventricular dyssynchrony was assessed using the Yu index, defined as the standard deviation of the time between the onset of QRS and the peak systolic velocity of tissue Doppler for 12 LV segments (six basal and six middle) in apical triplane-mode (4-V probe) (13, 14).

**Figure 2:**
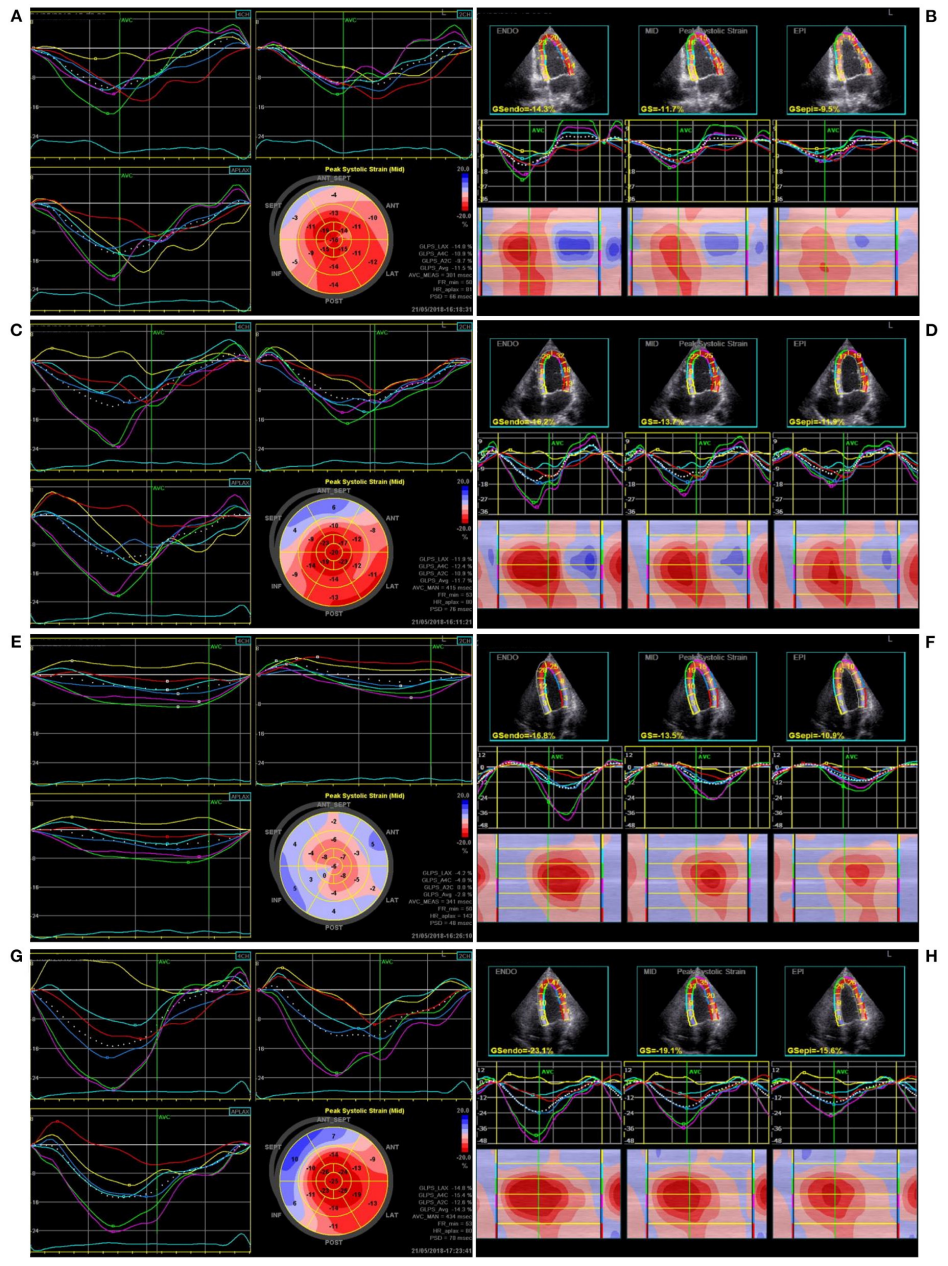
The ventricular global longitudinal peak strain and longitudinal layer-special strain observed in one patient during pacing at different sites. **(A,B)** Right ventricular apical pacing, **(C,D)** Right ventricular outflow tract pacing, **(E,F)** His bundle pacing, **(G,H)** Left bundle branch pacing.

The apical triplane-mode data were analyzed offline using an EchoPAC 203 workstation (GE Vingmed Ultrasound). The best cardiac cycle with good quality or clear endocardial boundaries was chosen, and the endocardial borders were automatically identified and tracked throughout the cardiac cycle. If the images were not optimal, manual adjustments were made. The LV wall of each apical view was divided into six segments. The global longitudinal peak strain (GLPS) (Figures 2A,C,E,G) and the standard deviation of the time to longitudinal peak strain of 17 segments (PSD) were automatically calculated. The longitudinal strain of the ventricular endocardium, mid-myocardium, and epicardium (Figures 2B,D,F,H) and the time to longitudinal peak strain (Ts) of the basal, middle, and apical segments of the lateral ventricular wall were simultaneously obtained. All echocardiograms were analyzed by an independent echocardiologist, blinded to the pacing modalities.

### Statistical Analyses

Continuous variables are described as means ± standard deviations; categorical variables are described as counts or percentages. When the data were or approximated normal distributions, comparisons among three or more conditions were evaluated using repeated measures one-way analysis of variance tests followed by the Tukey *post-hoc* analysis. Otherwise, the Friedman test was performed, and Dunn's *post-hoc* test was used to adjust the *P*-value. Statistical significance was defined as a two-sided *P* < 0.05. All statistical analyses were performed using GraphPad Prism 7.0 (GraphPad Software, San Diego, CA, USA).

## Results

### Baseline Characteristics

A total of 21 patients (15 men and 6 women) were enrolled in the study, with a mean age of 66.1 ± 13.0 years. All procedures were successfully performed in these patients. Of these, 19 patients were diagnosed with AVB, including one with first-degree AVB, 10 with second-degree AVB, and eight with third-degree AVB, and two with SSS. The mean heart rate was 53.8 ± 16.2 beats/min and the mean QRS duration was 118.8 ± 24.6 ms at the baseline ECG (Table 1).

**Table 1:** Baseline characteristics of patients.

	**Patients (*n* = 21)**
Age (years)	66.1 ± 13.0
Gender (male, *n*)	15 (71%)
Heart rate (beats/min)	53.8 ± 16.2
QRS duration (ms)	118.8 ± 24.6
First-degree AVB with AF (*n*, %)	1 (5%)
Second-degree AVB (*n*, %)	10 (47%)
Third-degree AVB (*n*, %)	8 (38%)
SSS (*n*, %)	2 (10%)

*Data are presented as mean ± standard (SD) for continuous variables, and number of subjects (n) and percentage (%) for categorical variables. AF, Atrial flutter; AVB, Atrioventricular block; SSS, Sick sinus syndrome*.

### Cardiac Systolic Function

To compare the acute changes in cardiac contraction between the different pacing sites, we measured the LVEDV, LVESV, LVEF, and TAPSE. We also evaluated the GLPS and the longitudinal layer-specific myocardial strains of the LV and RV [endocardium (endo), mid-myocardium (mid), and epicardium (epi): LVendo, LVmid, LVepi, RVendo, RVmid, and RVepi] in the apical four-chamber view using EchoPAC 203 (Table 2). LVEDV, LVESV, LVEF, GLPS, and LV strains were used to evaluate left ventricle systolic function, whereas TAPSE and RV strains for right ventricle systolic function.

**Table 2:** Comparison of the acute change of different pacing sites on cardiac contraction.

	**Baseline**	**RVA**	**RVOT**	**HIS**	**LBB**
LVEDV (mL)	79.0 ± 20.7	68.6 ± 22.9	69.1 ± 24.5	66.0 ± 20.9	64.6 ± 19.5^*^
LVESV (mL)	27.0 ± 9.7	28.8 ± 14.0	29.4 ± 15.8	25.7 ± 12.9	24.6 ± 10.8
LVEF (%)	65.6 ± 7.0	59.5 ± 8.8^*^	58.8 ± 9.4^*^	62.7 ± 6.9	62.8 ± 5.3
TAPSE (mm)	21.2 ± 3.4	17.4 ± 2.8^*^	17.6 ± 3.0^*^	19.4 ± 2.6^#^	19.1 ± 2.7^#^
GLPS (%)	−20.1 ± 4.7	−13.1 ± 4.1^*^	−14.1 ± 4.0^*^	−14.2 ± 3.9^*^	−14.9 ± 3.2^*^
LVendo (%)	−21.5 ± 3.3	−16.7 ± 4.9^*^	−16.6 ± 5.1^*^	−16.3 ± 4.3^*^	−19.4 ± 4.1
LVmid (%)	−18.7 ± 2.8	−14.3 ± 4.3^*^	−14.2 ± 4.3^*^	−14.3 ± 3.8^*^	−16.5 ± 3.6
LVepi (%)	−16.3 ± 2.5	−12.4 ± 3.8^*^	−12.0 ± 3.6^*^	−12.4 ± 3.4^*^	−14.3 ± 3.1
RVendo (%)	−21.2 ± 5.0	−16.0 ± 5.6^*^	−16.7 ± 4.4^*^	−16.3 ± 5.7^*^	−17.0 ± 3.8^*^
RVmid (%)	−18.8 ± 4.6	−14.1 ± 5.6^*^	−14.4 ± 4.1^*^	−14.0 ± 5.4^*^	−14.5 ± 3.5^*^
RVepi (%)	−16.8 ± 4.4	−12.7 ± 5.9^*^	−12.7 ± 3.8^*^	−12.3 ± 5.3^*^	−12.7 ± 3.2^*^

*Values are mean ± (SD). RVA, right ventricular apex; RVOT, right ventricular outflow tract; HIS, His bundle; LBB, left bundle branch; LVEDV, left ventricular end-diastolic volume; LVESV, left ventricular end-systolic volume; LVEF, left ventricular ejection fraction; TAPSE, tricuspid annular plane systolic excursion; GLPS, global longitudinal peak strain; The longitudinal strain of left ventricular endocardium (LVendo), mid-myocardium (LVmid) and epicardium (LVepi) were measured in apical four chamber view; The longitudinal strain of right ventricular endocardium (RVendo), mid-myocardium (RVmid) and epicardium (RVepi) were measured in apical four chamber view*.

* *P < 0.05 vs. baseline*,

# *P < 0.05 vs. RVA*.

#### Echocardiography Parameters

**Figure 3:**
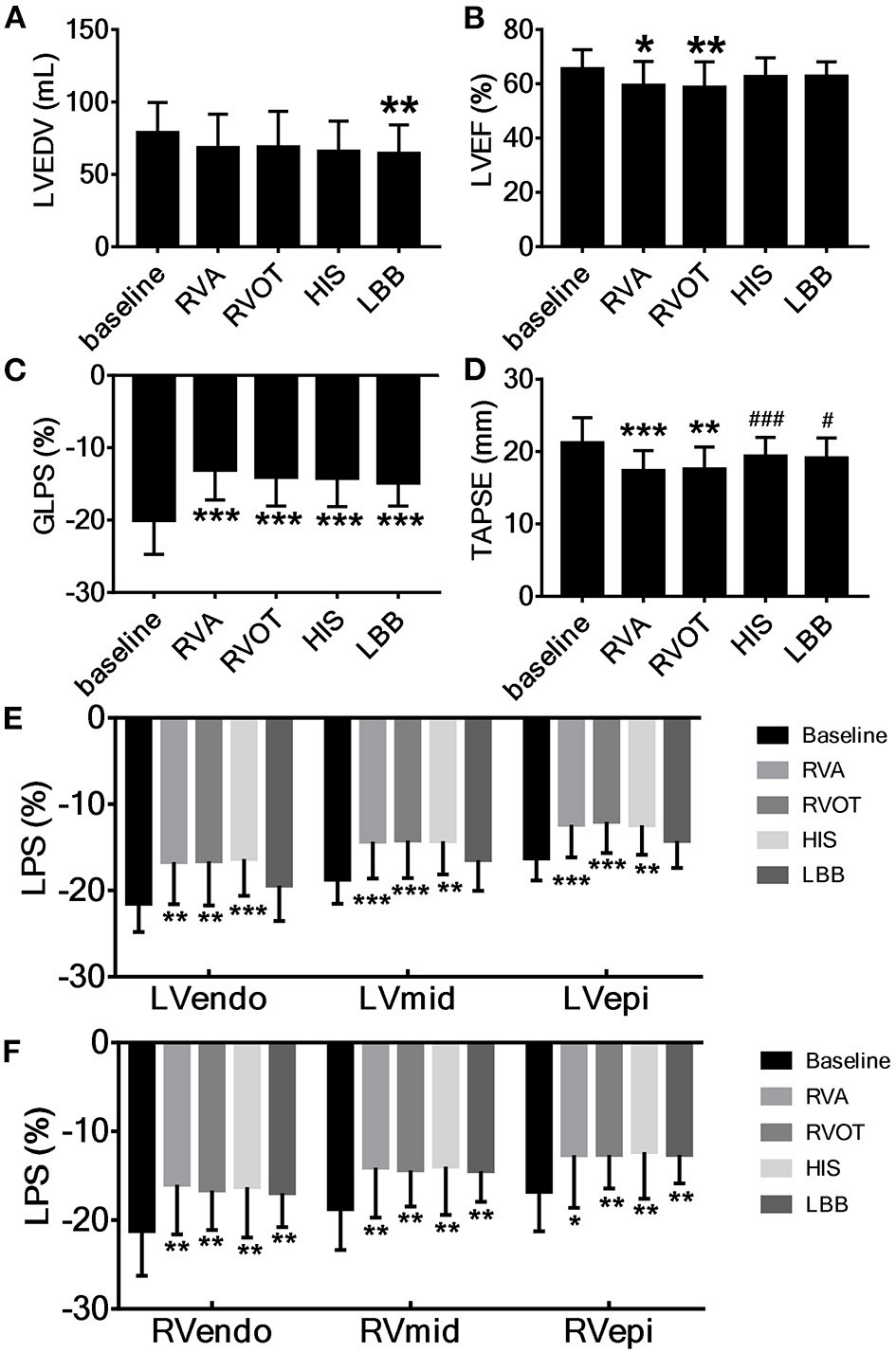
Acute change in cardiac contraction associated with pacing at the different sites. **(A)** LVEDV, left ventricular end-diastolic volume, **(B)** LVEF, left ventricular ejection fraction, **(C)** GLPS, global longitudinal peak strain, **(D)** TAPSE, tricuspid annular plane systolic excursion. **(E)** The longitudinal strain of left ventricular endocardium (LVendo), mid-myocardium (LVmid) and epicardium (LVepi). **(F)** The longitudinal strain of right ventricular endocardium (RVendo), mid-myocardium (RVmid) and epicardium (RVepi). ^*^P < 0.05, ^**^P < 0.01, ^***^P < 0.001 vs. baseline, ^#^*P* < 0.05, ^###^*P* < 0.001 vs. right ventricular apex (RVA).

The LVEDV during LBBP was significantly smaller than at baseline (*p* < 0.01, Figure 3A), whereas the LVESV was not significantly different across the various pacing sites. The mean LVEF was significantly lower during RVAP [59.5 ± 8.8% (*p* < 0.05)] and RVOP [58.8 ± 9.4% (*p* < 0.01)] than at baseline (65.6 ± 7.0%) (Figure 3B). Compared with baseline, the TAPSE during RVAP (*p* < 0.001) and RVOP (*p* < 0.01) were significantly reduced (Figure 3D); however, the TAPSE during HBP and LBBP had no significant difference. In addition, the TAPSE during HBP and LBBP were significantly higher than during RVAP (*p* < 0.001 and *p* < 0.05, respectively; Figure 3D).

#### Strain

At all pacing sites, the absolute values of GLPS (*p* < 0.001, Figure 3C), RVendo, RVmid, and RVepi were significantly lower than at baseline (Figure 3F). Except for LBBP, the absolute values of LVendo, LVmid, and LVepi at the other three pacing sites were also significantly lower than at baseline (Figure 3E).

### Cardiac Synchrony

We analyzed heart rate (HR) and QRS duration using ECG; IVMD, PSD, and the Yu index were analyzed by echocardiography. Moreover, we also measured the Ts of the apical, middle, and basal segments of the lateral wall of left ventricle (Lat_ap, Lat_mid, Lat_bas, respectively) or right ventricle (RV_ap, RV_mid, RV_bas, respectively) in the four-chamber view, as well as the difference in Ts between the basal, middle, and apical segments of left and right ventricle lateral walls (LV-RV_bas, LV-RV_mid, LV-RV_ap, respectively; Table 3).

**Table 3:** Comparison of the acute effect of different pacing sites on cardiac synchrony.

	**Baseline**	**RVA**	**RVOT**	**HIS**	**LBB**
HR (beats/min)	53.8 ± 16.2	78.8 ± 13.4^*^	79.0 ± 13.2^*^	78.6 ± 9.7^*^	77.8 ± 9.1^*^
QRS duration (ms)	118.8 ± 24.6	160.7 ± 24.7^*^	140.9 ± 13.9^*, #^	114.8 ± 18.2^#, Δ^	116.2 ± 11.6^#, Δ^
IVMD (ms)	3.1 ± 23.1	32.0 ± 30.5^*^	22.6 ± 21.4	1.0 ± 21.1^#, Δ^	−14.9 ± 28.3^#, Δ^
PSD (ms)	52.6 ± 17.4	70.3 ± 17.7^*^	62.2 ± 18.9	62.0 ± 19.7	58.6 ± 16.8
Yu index (ms)	57.6 ± 28.7	66.9 ± 33.2	63 ± 33.9	51.6 ± 25.0	44.5 ± 21.9^#^
Lat_ap (ms)	387.3 ± 46.9	357.2 ± 58.9	358.8 ± 50.9	403.9 ± 58.8	353.3 ± 63.3
Lat_mid (ms)	411.5 ± 53.1	382.9 ± 59.4	396.7 ± 62.8	404.0 ± 90.0	377.2 ± 68.4
Lat_bas (ms)	431.2 ± 71.3	411.3 ± 53.1	419.7 ± 68.9	408.8 ± 95.5	396.6 ± 74.1
RV_bas (ms)	369.8 ± 49.6	307.4 ± 97.3	307.0 ± 77.9	373.9 ± 97.5	330.7 ± 76.8
RV_mid (ms)	366.4 ± 49.8	302.0 ± 92.4	314.1 ± 83.0	379.8 ± 66.3	334.1 ± 55.1
RV_ap (ms)	380.5 ± 45.1	356.1 ± 56.7	349.4 ± 77.5	409.6 ± 87.4	374.1 ± 84.5
LV-RV_bas (ms)	61.4 ± 83.0	93.3 ± 130.8	112.0 ± 73.8	34.8 ± 117.7	67.5 ± 94.3
LV-RV_mid (ms)	45.1 ± 75.7	71.3 ± 118.3	63.6 ± 69.0	24.2 ± 85.8	33.6 ± 67.0
LV-RV_ap (ms)	6.8 ± 55.0	−6.6 ± 90.3	16.6 ± 65.4	−5.9 ± 85.2	−23.4 ± 89.6

*Values are mean ± (SD). RVA, right ventricular apex; RVOT, right ventricular outflow tract; HIS, His bundle; LBB, left bundle branch; IVMD, interventricular mechanical delay; PSD, the standard deviation of time to longitudinal peak strain of 17 segments; Yu index, the standard deviation of time from QRS to peak systolic velocity in ejection phase for 12 LV segments; Lat_ap, Lat_mid, Lat_bas, the time to peak longitudinal strain of apical (ap), middle (mid) and basal (bas) segment of lateral (Lat) wall of left ventricle in four chamber view; RV_bas, RV_mid, RV_ap, the time to peak longitudinal strain of apical (ap), middle (mid) and basal (bas) segment of lateral wall of right ventricle (RV) in four chamber view; LV-RV_bas, LV-RV_mid, LV-RV_ap, The difference of the time to peak longitudinal strain between basal (or middle or apical) segment of left ventricular and right ventricular lateral wall; HR, heart rate*.

* *P < 0.05 vs. baseline*,

# *P < 0.05 vs. RVA*,

Δ *P < 0.05 vs. RVOT*.

#### Electrical Synchrony

**Figure 4:**
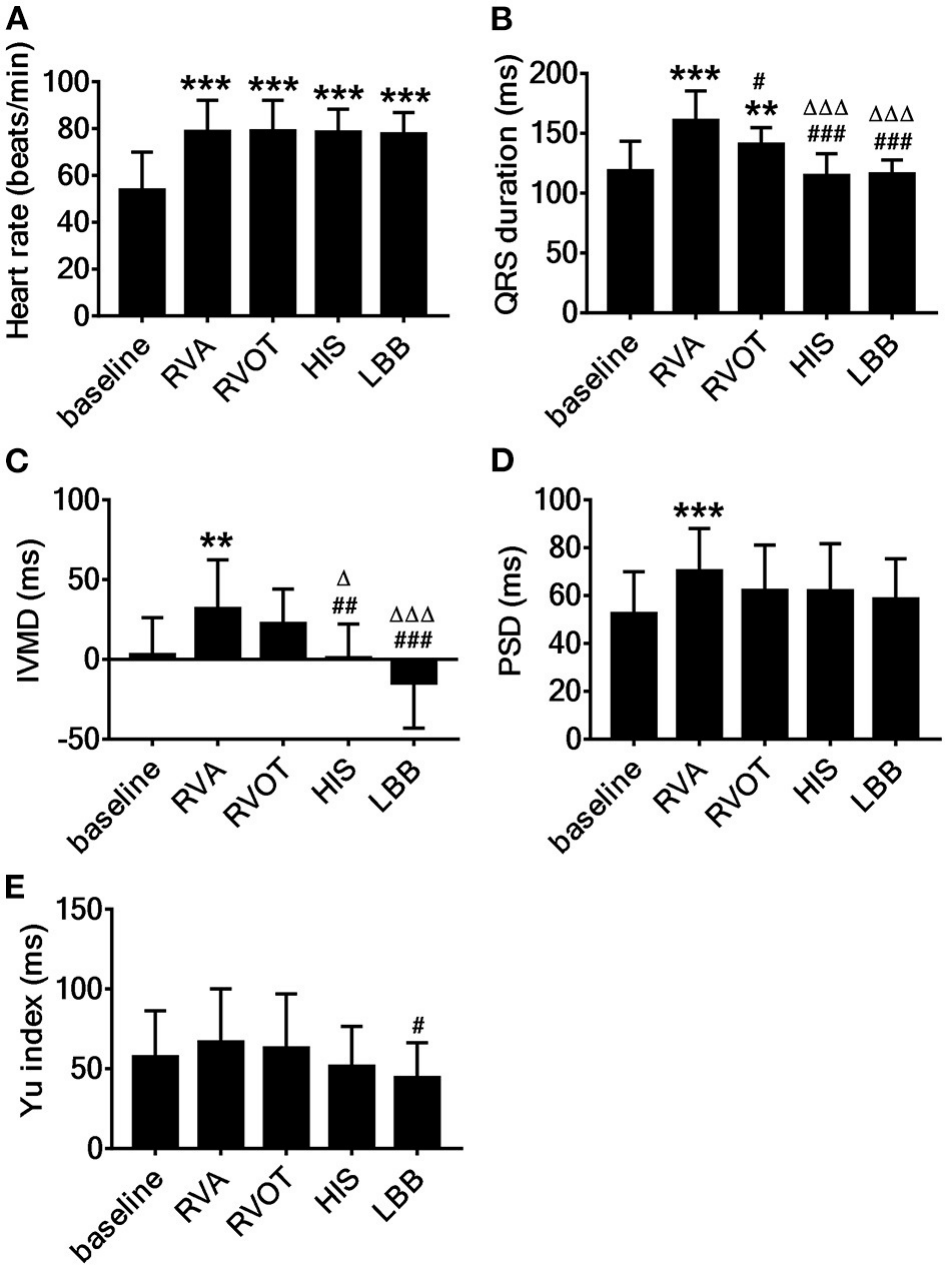
Acute effect on cardiac synchrony associated with pacing at the different sites. **(A)** Heart rates, **(B)** QRS duration, **(C)** IVMD, interventricular mechanical delay, **(D)** PSD, the standard deviation of time to longitudinal peak strain of 17 segments, **(E)** Yu index, the standard deviation of time from QRS to peak systolic velocity in ejection phase for 12 left ventricular segments. ^**^P < 0.01, ^***^P < 0.001 vs. baseline, ^#^*P* < 0.05, ^##^*P* < 0.01, ^###^*P* < 0.001 vs. right ventricular apex (RVA), ^Δ^*P* < 0.05, ^ΔΔΔ^*P* < 0.001 vs. right ventricular outflow tract (RVOT).

The mean HR at baseline was 53.8 ± 16.2 beats/min. The HR at four pacing sites was significantly increased (*p* < 0.001, Figure 4A). The mean QRS duration at baseline was 118.8 ± 24.6 ms but was significantly longer during RVAP and RVOP (*p* < 0.001, *p* < 0.01, respectively; Figure 4B); the mean QRS durations during HBP and LBBP had no significant difference (Figure 4B). Compared with RVAP, RVOP had a smaller effect on QRS duration (*p* < 0.05, Figure 4B). HBP and LBBP had significantly narrower QRS durations (*p* < 0.001 and *p* < 0.001, respectively; Figure 4B) compared with RVAP or RVOP.

#### Interventricular Mechanical Dyssynchrony

The mean IVMD during RVAP was 32.0 ± 30.5 ms and was significantly longer than at baseline (3.1 ± 23.1 ms, *p* < 0.01; Figure 4C). There was no significant difference in IVMD between baseline and either HBP or LBBP (Figure 4C). Meanwhile, the IVMD during HBP and LBBP were significantly shorter than during RVAP (*p* < 0.01 and *p* < 0.001, respectively; Figure 4C) or RVOP (*p* < 0.001, *p* < 0.01 respectively, Figure 4C). The PSD was significantly larger during RVAP than at baseline (*p* < 0.001; Figure 4D) but was not significantly different from baseline during RVOP, HBP, or LBBP (Figure 4D). To explore the local synchrony of the ventricles, we compared the Ts of three segments (LV-RV_bas, LV-RV_mid, LV-RV_ap) and failed to find significant differences.

#### Intraventricular Mechanical Dyssynchrony

The mean Yu index was 57.6 ± 28.7 ms at baseline, 66.9 ± 33.2 ms during RVAP, 63 ± 33.9 ms during RVOP, 51.6 ± 25.0 ms during HBP and 44.5 ± 21.9 ms during LBBP. The Yu index during LBBP was significantly shorter than during RVAP (*p* < 0.05; Figure 4E).

## Discussion

Pacemaker implantation is necessary for patients with a high degree AVB, where various pacing modalities can be chosen according to their respective advantages. The RVA and RVOT are conventional pacing sites because of their stability and ease of pacemaker implantation. However, previous studies have reported that RVAP increases the mortality and hospitalization rates of patients with heart failure (15) and does not alleviate cardiac valvular regurgitation or improve long-term clinical outcomes (16). The stimulus for RV pacing (RVP) must pass through the myocardial tissue first and then reach the conduction system, extending the activation time of the left ventricle. The conduction sequence during RVAP is contrary to that of the normal sequence. These limitations lead to cardiac electrical dyssynchrony and regional cardiac contraction discordance, ultimately causing ventricular mechanical dyssynchrony. Thus, physiological pacing is urgently required to maintain normal electrical conduction and achieve cardiac electrical and mechanical synchrony. HBP is considered an ideal physiological pacing mode duo to the relatively normal sequence of ventricular electrical activation and ventricular contraction synchrony, leading to better hemodynamics. In 2018, the American College of Cardiology, American Heart Association, and American Heart Rhythm Society jointly published guidelines for the evaluation and management of patients with bradycardia and cardiac conduction delay, and included HBP for the first time (17). Recently, LBBP has attracted broad interest as a new physiological pacing modality. In the present study, RVAP, RVOP, HBP, and LBBP were performed in the same patients and the acute effects on cardiac synchrony and contraction of pacing at these sites were compared. To a certain extent, the acute effects on cardiac synchrony can help predict long-term outcomes. Patients who had acute deteriorated LV synchrony after cardiac resynchronization therapy (CRT) demonstrated worse outcomes than those who had improved LV synchrony (18).

### Cardiac Systolic Function

Many studies have investigated the feasibility, safety, and clinical outcomes of HBP. Sharma et al. (8) attempted HBP in 94 patients, and succeeded in 75(80%). They found that the HBP group required longer implantation times and a higher pacing threshold than the RVP group (98 patients). Heart failure hospitalization was significantly reduced in patients with >40% ventricular pacing in the HBP group than in the RVP group. For patients with no response to CRT or failure of LV electrode implantation, HBP corrected basal conduction disturbances and improved echocardiographic measurements as an alternative treatment for CRT (19). HBP was also employed to control atrial fibrillation in patients with heart failure who underwent atrioventricular node ablation, significantly improving their LVEF and NYHA classification (20). Our results provide complementary information with previous findings that showed that LVEF and TAPSE deteriorated during RVAP and RVOP, but had little influence on HBP and LBBP; LBBP evidently improved LVEDV. We measured GLPS and longitudinal layer-specific myocardial strains to accurately evaluate the regional mechanical motion of the ventricular myocardium. Although the longitudinal layer-specific strains of LV and RV and GLPS were significantly decreased at all pacing sites as the HR was corrected to within the normal range, LBBP showed the least impact. These results indicate that HBP and LBBP best maintained cardiac contraction. However, HBP has several limitations (21), including the requirement for skilled operation due to the difficulty in locating the His bundle and having a high pacing threshold and low sense. It is also not applicable to blocks below His bundle or to diffuse ventricular blocks caused by myocardial disease. Moreover, HBP cannot provide protection when cardiac conduction system lesions deteriorate. Hence, the investigation of new LV pacing (LVP) sites is required. In 2003, Peschar et al. (22) first conducted LVP in anesthetized, open-chest dogs with normal ventricular conduction. The immediate results demonstrated better maintained LV pump functioning associated with the LVP sites than with RVP sites. Mills et al. (23) carried out LVP in dogs after atrioventricular nodal ablation and further verified that LVP was superior to RVP in chronically maintaining LV contractile coordination and pump function. LV septal pacing was first clinically applied in 2016 and showed better hemodynamic effects than RVP (24). In 2017, Huang et al. (4) successfully implemented the first LBBP in a heart failure patient with LBBB, and the cardiac function of the patient improved during 1 year of follow-up. Previous studies have reported that RBBB can be corrected during LBBP. Li et al. observed a narrowing of the complete RBBB morphology using unipolar LBBP at a high output (25). Sometimes bipolar pacing (26) or adjusting atrioventricular delay (4) can also correct incomplete RBBB. In this study, LBBP did not induce RBBB and did not influence cardiac hemodynamics. However, further studies with long-term follow-up are necessary.

### Cardiac Synchrony

Cardiac synchrony is essential for cardiac structure and function, and cardiac resynchronization can reverse LV remodeling and reduce the risk of heart failure events (27, 28). Pastore et al. (29) performed permanent HBP in 37 patients with normal cardiac function and added an RVA backup lead in each patient. Compared with HBP, RVAP resulted in a wider QRS duration, significantly longer LV isovolumetric contraction and relaxation times, and higher pulmonary arterial systolic pressure. In this study, we evaluated electrical and mechanical synchrony at four pacing sites. The QRS duration is the main index used to evaluate electrical synchrony; its normal value is <120 ms. RVAP and RVOP significantly prolonged the QRS duration, which did not change and remained within the normal range during HBP and LBBP. Therefore, RVAP and RVOP caused electrical dyssynchrony; HBP and LBBP preserved physiological electrical synchrony.

Currently, multiple methods are utilized to evaluate mechanical synchrony. For example, Zhang et al. (30) adopted gated, single-photon emission computed tomography (SPECT) myocardial perfusion imaging to study the LV mechanical synchrony associated with HBP and found that HBP resulted in better LV mechanical synchrony parameters. However, SPECT is a procedure that involves radiation and cannot be performed in the catheterization laboratory or at the bedside. Echocardiography is accurate and convenient for measuring cardiac contraction and hemodynamics in real-time without radiation exposure. So far, little is known about the acute outcomes on cardiac mechanical synchrony during LBBP, and comparisons among four different pacing sites are rare. In this study, the IVMD, PSD, and Yu index were used to evaluate inter- and intraventricular synchrony. We found that RVAP distinctly extended IVMD and PSD, and the Yu index tended to deteriorate during RVAP, suggesting that RVAP causes inter- and intraventricular dyssynchrony. LBBP significantly improved the IVMD and Yu index compared with RVAP. Among the four pacing modalities, RVAP resulted in the most unfavorable acute impact on mechanical synchrony, whereas HBP and LBBP had little influence on mechanical synchrony. LBBP represented the best physiological pacing mode and maintained ventricular synchrony.

This study was limited by its small sample size. Further, we only measured the immediate changes in myocardial mechanics after implantation of pacemaker electrodes. The long-term effects of pacing at each site require further investigation.

## Conclusion

Our study compared acute changes in cardiac synchrony and contraction among four pacing modalities (RVAP, RVOP, HBP, and LBBP) in the same patients (each with AVB or SSS), to evaluate the effect of the His-Purkinje system pacing on ventricular electrical and mechanical synchrony. Echocardiographic parameters including LVEF, GLPS, TAPSE, IVMD, PSD, and Yu index, provided more detailed evaluations of ventricular synchrony and contraction at different pacing sites than QRS duration. HBP and LBBP demonstrated similar added value in preserving physiological hemodynamics and cardiac function, implying their interchangeability under some conditions. Our results showed that LBBP could maintain cardiac hemodynamics similar to or better than HBP, providing more evidence for this alternative of physiological pacing modality. In conclusion, our study suggests that LBBP is an effective physiological pacing mode as HBP, which preserved normal cardiac contraction and synchrony.

## Data Availability Statement

The raw data supporting the conclusions of this article will be made available by the authors, without undue reservation.

## Ethics Statement

The studies involving human participants were reviewed and approved by the Ethics Committee of Zhongshan Hospital Affiliated to Fudan University. The patients/participants provided their written informed consent to participate in this study.

## Author Contributions

XS and HC contributed to the research design, analyzed the data, and critically revised the manuscript. XC contributed to the research design. HX contributed to the data collection, analyzed the data, and drafted the paper. YW contributed to the collection and analyzed the data. YC, YZ, and YaL contributed to the data interpretation. YuL and ZG contributed to critically revised the manuscript. All authors read and approved the final manuscript.

## Funding

This study was funded by National Natural Science Foundation of China (No. 82071933) and Shanghai Science, Technology Commission Science and technology innovation plan (No. 20JC1418400), 2018 Clinical Trial Funding of Zhongshan Hospital, Fudan University (No. 2018ZSLC14), and Youth Foundation of Shanghai Municipal Health Commission (No. 20194Y0272).

## Conflict of Interest

The authors declare that the research was conducted in the absence of any commercial or financial relationships that could be construed as a potential conflict of interest.

## Publisher's Note

All claims expressed in this article are solely those of the authors and do not necessarily represent those of their affiliated organizations, or those of the publisher, the editors and the reviewers. Any product that may be evaluated in this article, or claim that may be made by its manufacturer, is not guaranteed or endorsed by the publisher.

## Abbreviations

RVAP: Right ventricular apical pacing
RVOP: Right ventricular outflow tract pacing
HBP: His bundle pacing
LBBP: Left bundle branch pacing
RBBB: Right bundle branch block
AVB: Atrioventricular block
SSS: Sick sinus syndrome
ECG: Electrocardiogram
LVEDD: Left ventricular end-diastolic dimension
LVESD: Left ventricular end-systolic dimension
TAPSE: Tricuspid annulus plane systolic excursion
LVEF: Left ventricular ejection fraction
IVMD: Interventricular mechanical delay
GLPS: Global longitudinal peak strain
PSD: Standard deviation of time to longitudinal peak strain of 17 segments
Ts: Time to longitudinal peak strain
LVendo: Left ventricular endocardium
LVmid: Left ventricular mid-myocardium
LVepi: Left ventricular epicardium
RVendo: Right ventricular endocardium
RVmid: Right ventricular mid-myocardium
RVepi: Right ventricular epicardium
Lat_ap: the Ts of apical segment of lateral wall of left ventricular
Lat_mid: the Ts of middle segment of lateral wall of left ventricular
Lat_bas: the Ts of basal segment of lateral wall of left ventricular
RV_ap: the Ts of apical segment of lateral wall of right ventricular
RV_mid: the Ts of middle segment of lateral wall of right ventricular
RV_bas: the Ts of basal segment of lateral wall of right ventricular
LV-RV_bas: the difference of the Ts between basal segments of left ventricular and right ventricular lateral wall
LV-RV_mid: the difference of the Ts between middle segments of left ventricular and right ventricular lateral wall
LV-RV_ap: the difference of the Ts between apical segments of left ventricular and right ventricular lateral wall
RVP: Right ventricular pacing
LVP: Left ventricular pacing
CRT: cardiac resynchronization therapy.
